# Plasma Metabolomics of Acute Coronary Syndrome Patients Based on Untargeted Liquid Chromatography–Mass Spectrometry

**DOI:** 10.3389/fcvm.2021.616081

**Published:** 2021-05-20

**Authors:** Wei Zhong, Qiaoting Deng, Xunwei Deng, Zhixiong Zhong, Jingyuan Hou

**Affiliations:** ^1^Center for Cardiovascular Diseases, Meizhou People's Hospital (Huangtang Hospital), Meizhou Hospital Affiliated to Sun Yat-sen University, Meizhou, China; ^2^Guangdong Provincial Engineering and Technology Research Center for Molecular Diagnostics of Cardiovascular Diseases, Meizhou, China; ^3^Guangdong Provincial Key Laboratory of Precision Medicine and Clinical Translational Research of Hakka Population, Meizhou, China; ^4^Research Experimental Center, Meizhou People's Hospital (Huangtang Hospital), Meizhou Hospital Affiliated to Sun Yat-sen University, Meizhou, China

**Keywords:** acute coronary syndromes, metabolomics, diagnosis, biomarkers, liquid-chromatography coupled with tandem mass spectrometry

## Abstract

**Background:** Acute coronary syndrome (ACS) is the main cause of death and morbidity worldwide. The present study aims to investigate the altered metabolites in plasma from patients with ACS and sought to identify metabolic biomarkers for ACS.

**Methods:** The plasma metabolomics profiles of 284 ACS patients and 130 controls were carried out based on an untargeted liquid chromatography coupled with tandem mass spectrometry (LC-MS) approach. Multivariate statistical methods, pathway enrichment analysis, and univariate receiver operating characteristic (ROC) curve analysis were performed.

**Results:** A total of 328 and 194 features were determined in positive and negative electrospray ionization mode in the LC-MS analysis, respectively. Twenty-eight metabolites were found to be differentially expressed, in ACS patients relative to controls (*p* < 0.05). Pathway analysis revealed that these metabolites are mainly involved in synthesis and degradation of ketone bodies, phenylalanine metabolism, and arginine and proline metabolism. Furthermore, a diagnostic model was constructed based on the metabolites identified and the areas under the curve (AUC) for 5-oxo-D-proline, creatinine, phosphatidylethanolamine lyso 16:0, and LPC (20:4) range from 0.764 to 0.844. The higher AUC value of 0.905 was obtained for the combined detection of phosphatidylethanolamine lyso 16:0 and LPC (20:4).

**Conclusions:** Differential metabolic profiles may be useful for the effective diagnosis of ACS and may provide additional insight into the molecular mechanisms underlying ACS.

## Introduction

Despite a variety of available therapeutic options, coronary artery disease (CAD) remains a leading cause of mortality worldwide (1, 2). Atherosclerosis has a major pathogenic role for the progression of atherosclerotic coronary artery lesions. Subsequent atherosclerotic plaque rupture or disruption of the overlying endothelial surface is generally associated with obstructive CAD and thus predominantly leads to a wide range of acute coronary syndromes (ACS) (3–5). Rapid and early detection of ACS is an urgent clinical need, as delays increase the risk of morbidity and mortality. Circulating biomarkers within the blood stream are measurable and quantifiable biological parameters that possess a big diagnostic potential serve as indices for physiological and pathophysiological assessments of ACS (6–8). The study of novel biomarkers for the screening and diagnosis of ACS has been the focus of extensive research, but it is still not fully understood.

Recent advances in genomics, proteomics, and other omics technologies led to the innovative development of alternative biomarkers that can facilitate earlier diagnosis of diseases (9, 10). Metabolomics has emerged as a powerful tool for small-molecule identification and quantification through using various metabolomics detection methods (11, 12). Indeed, the small molecules are directly involved in cell composition and metabolism, which might be related to disease-specific biochemical changes and may provide key information for the state of the organism (13, 14). Analytical methods have improved tremendously, enabling the identification of metabolite alterations at the molecular level in biological samples and thereby characterizing aberrant physiological status (15, 16). In the past decade, human metabolomics has been extensively exercised for understanding the fine-tuning of comprehensive metabolic profiling that is associated with different kinds of diseases, such as cancer, Alzheimer, diabetes, and renal disease (17–22).

To date, interest has emerged on the role of differential plasma metabolite occurred in the development of various types of CAD (23–25). Metabolomics is a promising tool of cardiovascular biomarker discovery. Several recent studies have investigated the metabolic profiles in CAD and a fraction of significant altered metabolites have been identified such as amino acids, fatty acid, and several lipid classes, which have been associated with atherosclerosis development (26–29). Despite the progress made, the relevant findings and conclusions of the metabolic signature in ACS are very scarce.

In the present study, to gain a deeper insight into atherosclerosis-related ACS, we first used the untargeted liquid chromatography coupled with tandem mass spectrometry (LC-MS) metabolomics profiling approach to uncover the metabolite alterations in plasma samples from ACS patients. We highlight that novel and intriguing metabolites may serve as diagnostic biomarkers for the differential diagnosis of ACS and improve our understanding of the complex cardiac pathogenic mechanism of atherosclerosis.

## Materials and Methods

### Study Population

**Figure 1:**
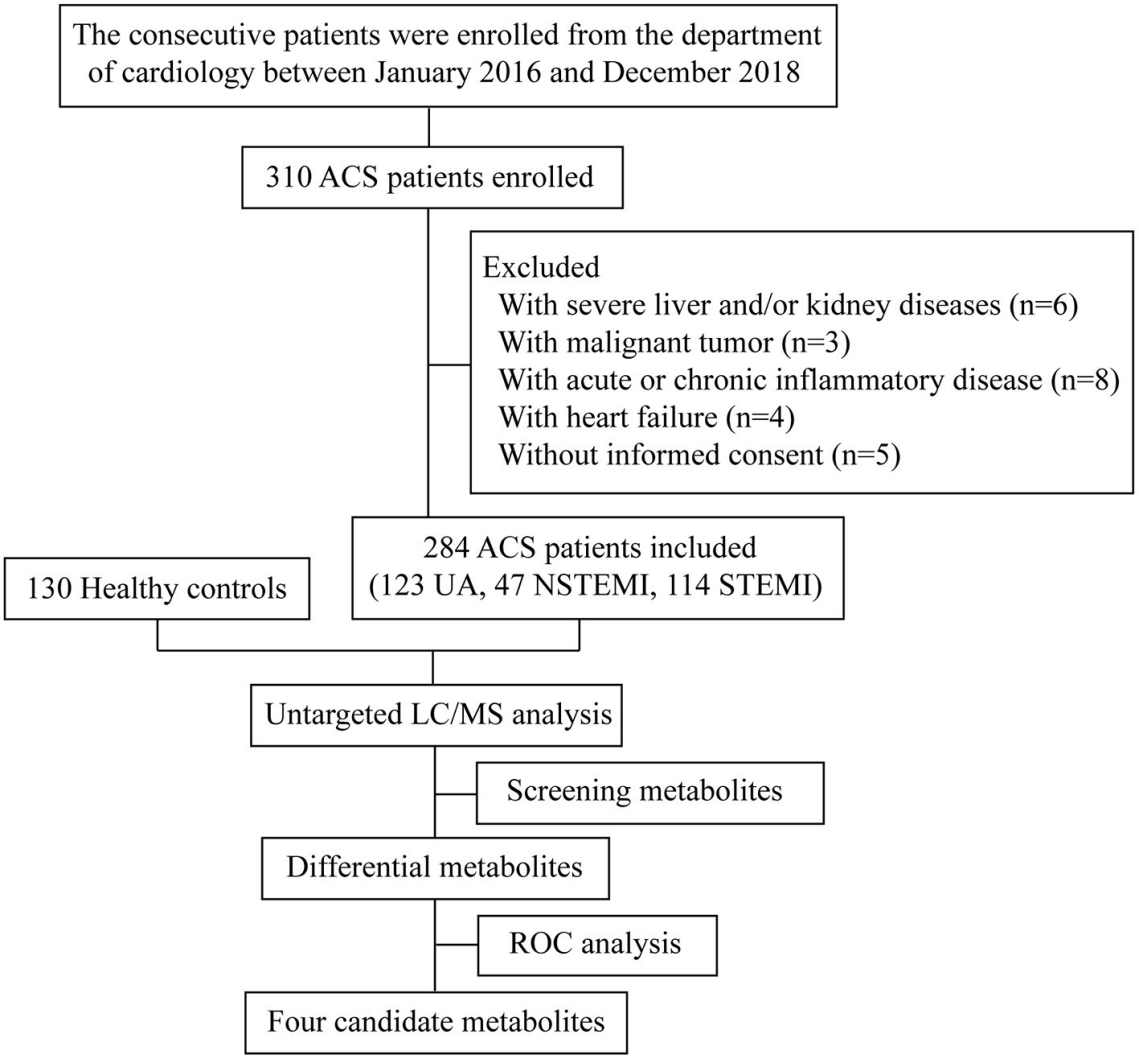
The study flowchart.

The consecutive patients were derived from the department of cardiology in our hospital between January 2016 and December 2018 in this study. Of these, 284 ACS patients with angiographically confirmed ≥50% stenosis of the left main coronary trunk or ≥70% stenosis in a major epicardium artery were included. All of the ACS patients were newly diagnosed and comprised of the following three clinical subtypes: non-ST-elevation myocardial infarction (NSTEMI), ST-elevation myocardial infarction (STEMI), and unstable angina (UA). The diagnoses of NSTEMI and STEMI were made by the electrocardiographic (ECG) changes as well as cardiac enzyme levels. The diagnosis of UA patients was made by the presence of angina pectoris together with evidence of ischemic ECG changes and no elevation in troponin. On the other hand, a total of 130 outpatients for health examination during the same period were selected as the healthy control group. ACS patients with severe liver and/or kidney diseases, malignant tumor, autoimmune disorders, acute or chronic inflammatory disease, and heart failure were ruled out in this study. The study flowchart is illustrated in Figure 1. The demographic and clinical data were collected retrospectively by a trained study coordinator based on a standardized protocol. The presented study was approved by the Medical Ethics Committee of the Meizhou People's Hospital and complied with the principles of Declaration of Helsinki. All study participants included gave written informed consent.

### Sample Preparation for Metabolomics

Whole-venous blood samples were drawn in plastic EDTA-coated tubes from all subjects on admission and centrifuged at 3,000 rpm, at 4°C for 10 min. The supernatant plasma was aliquoted and stored at −80°C to prevent sample degradation. Hemolytic samples were excluded. Prior to metabolomics analysis, frozen samples were taken out at −80°C and acclimated at room temperature. An aliquot of 100 μl plasma sample was transferred into 1.5-mL polypropylene tubes and precipitated with 200 μl of precooled methanol. Thereafter, samples were vortexed for 60 s and then centrifuged at 12,000 rpm, at 4°C for 10 min. Subsequently, the supernatant was carefully filtered using a 0.22-μm membrane and then pipetted directly into a 2-ml LC-MS autosampler vial. To ensure instrument stability and data quality for metabolic profiling, a quality control (QC) sample was generated by pooling 20 μL of all the plasma samples.

### Metabolomics Analysis by LC-MS

To obtain a comprehensive metabolic profile, untargeted metabolomics analysis for metabolic profiling was conducted using the Acquity UPLC system (Waters Corporation; Milford, MA, USA) connected to a Thermo LTQ-Orbitrap XL mass spectrometer (Thermo Fisher Scientific, Inc.; Waltham, MA, USA) equipped with an electrospray ionization (ESI) source. Chromatographic separation was performed in an Acquity UPLC system equipped with an Acquity UPLC BEH-C18 column (100 × 2.1 mm, 1.7 μm, Waters). Five microliters of each sample was injected after equilibration at a constant injector temperature of 40°C. The mobile phase was composed of A (0.1% formic acid, v/v) and B (acetonitrile) at a flow rate of 0.25 mL/min. A gradient of solvent B (v/v) from 2 to 98% was run over 15.0 min. The column was then re-equilibrated for 3 min for starting conditions. The ESI-MS^n^ experiment was executed as follows: The voltages of capillary and tube were 35 and 50 V, −15 and −50 V in positive and negative modes, respectively. The sheath gas flow rate was 45 arbitrary units (AU), auxiliary gas flow rate 15 AU, capillary temperature 325°C, and spray voltages of 4.8 and −4.5 kV. The Orbitrap analyzer was used in Fourier scanned transform mass spectrometry (FTMS) full MS scan mode. The MS data was collected which range from 50 to 1,000 m/z with a high mass resolution of 60,000. Collision-induced dissociation product ion mass spectra were conducted in data-dependent acquisition (DDA) MS/MS mode with a normalized collision energy set as 30 eV. Further, dynamic exclusion was set to a repeat count of 2 with a 15-s exclusion duration.

### Data Processing

The raw data were processed using the XCMS software, which incorporates non-linear retention time alignment, peak discrimination, filtering, alignment, matching, and identification (30). XCMS parameters were default settings except for the following: bw = 5, ppm = 15, peak width = c (10, 120), mzwid = 0.015, mzdiff = 0.01, method = “centWave.” The mass spectrometry data were converted to the mzXML format using ProteoWizard software (version 3.0). Subsequently, the resultant data matrices were fed to SIMCA-P software (Version 15.0, Umetrics AB, Umeå, Sweden) and R package RoPLS for multivariate principal component analysis (PCA), partial least-square discriminant analysis (PLS-DA), and orthogonal partial least-square discriminant analysis (OPLS-DA). Total explained variance (*R*^2^) and predictability (*Q*^2^) were performed to determine the validity of the models. Metabolites were identified by mass spectrum matching against reference spectra of the Human Metabolome Database (http://www.hmdb.ca), Metlin (http://metlin.scripps.edu), massbank (http://www.massbank.jp/), LipidMaps (http://www.lipidmaps.org), MONA (http://mona.fiehnlab.ucdavis.edu/) with a mass accuracy of 20 ppm, and database built by BioNovoGene Co., Ltd. A two-sided Student's *t*-test was applied to find the level of the significance between the metabolites. Variable importance in the projection (VIP) scores more than 1.0 and false discovery rate (FDR) value < 0.05 was used for the identification of potential metabolic biomarkers. Hierarchical cluster analysis (HCA) was performed using Cluster (version 3.0). Furthermore, the disturbed metabolites and their respective metabolic pathways were identified by using Kyoto Encyclopedia of Genes and Genomes (KEGG) pathway database (http://www.genome.jp/kegg/) and MetaboAnalyst (http://www.metaboanalyst.ca/).

### Statistical Analysis

All statistical analyses were performed with Statistical Package for the Social Sciences 19.0 software (SPSS, Inc., USA). Continuous variables were expressed as mean ± standard deviation. Categorical data were expressed as count and percentages. The comparison between the ACS patients and controls using Fisher's exact test or Student's *t*-tests as appropriate. Univariate receiver operating characteristic (ROC) curve analysis was plotted as a measure for assessing the clinical performance of metabolites, and the area under the curve (AUC) was assessed. *P*-value of <0.05 was considered statistically significant.

## Results

### Baseline Characteristics of the Study Population

The present study population comprised of 284 patients with newly diagnosed ACS (47 NSTEMI patients, 114 STEM patients, and 123 UA patients) and 130 health controls. The baseline characteristics and laboratory data of all the study population are shown in Table 1. There were no statistically significant differences in clinical parameters, such as systolic blood pressure (SBP), diastolic blood pressure (DBP), smoking, drinking, and hypercholesterolemia between the groups for ACS patients and controls (*p* > 0.05). Compared with the control group, ACS patients had significantly older age, higher proportion of males, higher prevalence of hypertension, and diabetes mellitus (*p* < 0.05). In addition, the ACS patients had higher low-density lipoprotein (LDL) levels and lower high-density lipoprotein (HDL) levels (*p* < 0.05). Other laboratory values such as triglyceride (TG), total cholesterol (TC), hemoglobin, and platelet count were not significantly different between groups (*p* > 0.05).

**Table 1:** Baseline characteristics of the study population.

**Characteristics**	**Control**	**ACS**	***p*-value**
	**(*n* = 130)**	**(*n* = 284)**	
Age (years)	61.99 ± 9.70	65.50 ± 11.05	0.001
Gender (%)	67 (52.3)	217 (76.4)	<0.001
SBP (mm Hg)	132.72 ± 20.79	135.16 ± 22.49	0.280
DBP (mm Hg)	80.89 ± 13.63	79.70 ± 13.80	0.415
Hypertension (%)	63 (48.46)	167 (58.80)	0.049
Diabetes mellitus (%)	18 (13.85)	107 (37.68)	<0.001
Smoking (%)	22 (16.92)	69 (24.30)	0.093
Drinking (%)	2 (1.54)	13 (4.58)	0.125
Hypercholesterolemia (%)	31 (23.85)	90 (31.69)	0.103
TG (mmol/L)	1.53 ± 1.19	1.72 ± 1.08	0.117
TC (mmol/L)	4.72 ± 1.19	4.85 ± 1.27	0.336
LDL-C (mmol/L)	2.57 ± 0.75	2.75 ± 0.94	0.035
HDL-C (mmol/L)	1.32 ± 0.36	1.20 ± 0.31	0.002
Hemoglobin (g/L)	134.48 ± 17.54	132.46 ± 19.15	0.308
Platelet count (×10^9^/ml)	221.56 ± 62.72	221.62 ± 73.44	0.994

*Data values are means ± standard deviation or numbers (%)*.

*SBP, systolic blood pressure; DBP, diastolic blood pressure; TG, triglyceride; TC, total cholesterol; LDL-C, low-density lipoprotein cholesterol; HDL-C, high-density lipoprotein cholesterol*.

### Metabolomics Analysis of Plasma Samples From ACS Patients and Control Subjects

**Figure 2:**
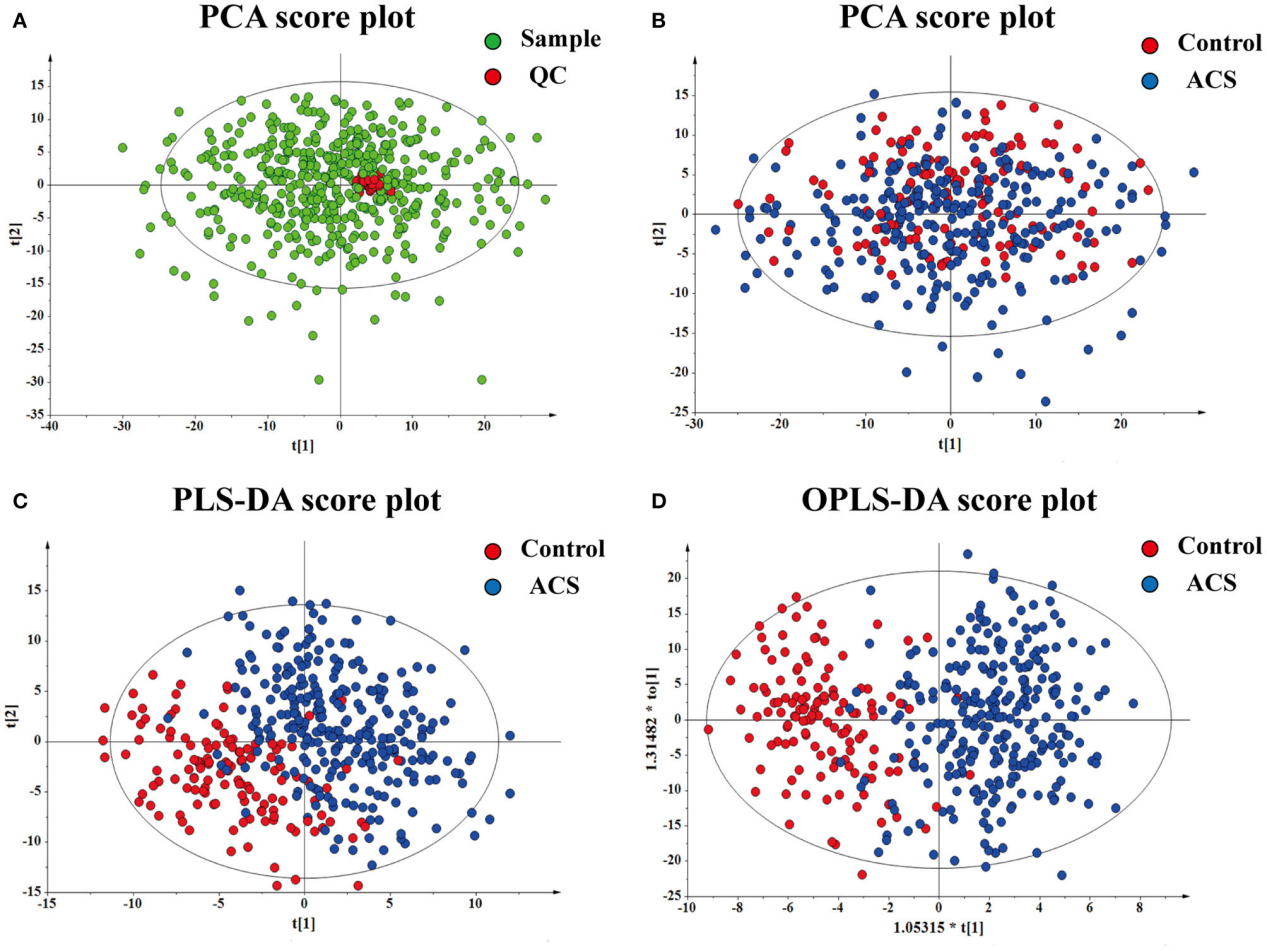
Results of multivariate statistical analysis for plasma metabolomics profiling. **(A)** PCA for the QC. **(B)** PCA for the sample. **(C)** PLS-DA for the sample. **(D)** OPLS-DA for the sample. Each dot represents a plasma sample.

Representative spectra of the identified metabolites obtained from untargeted LC-MS metabolomics profiling analysis are shown in Supplementary Figure 1. All data were normalized to the summed total ion intensity per chromatogram, and the resultant data matrices were introduced to SIMICA-P 15.0 software. Briefly, after the standard procedure in signal de-noising and dataset normalization, a total of 582 molecular features (328 negative-mode features and 194 positive-mode features) were identified for subsequent analyses. In this study, PCA was initially used to determine the reliability of the metabolic profiling method. The red dots indicated that the QC samples were center clustered, suggesting that the mass accuracy of the MS data fit the requirement (Figure 2A). PCA, PLS-DA, and OPLS-DA were carried out to determine whether it was possible to distinguish controls and ACS patients on the basis of the metabolomics data. PCA indicated that there was no discernible separation between the controls and ACS patients (*R*^2^X = 52.0%, *Q*^2^ = 24.5%) (Figure 2B). The statistical evaluation by the PLS-DA model showed a good separation of the two groups, implying distinct metabolic variations associated with ACS (*R*^2^Y = 69.6%, *Q*^2^ = 49.8%) (Figure 2C). Similarly, OPLS-DA score plots also display a distinct separation between these two groups (*R*^2^Y = 69.6%, *Q*^2^ = 44.3%) (Figure 2D). These results demonstrated the reliable differentiation in metabolic changes between ACS patients and controls.

### Differential Metabolite Identification and Pathway Analysis

**Figure 3:**
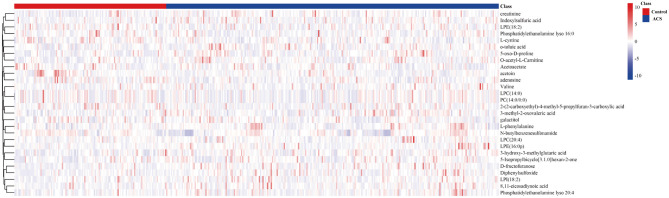
The hierarchical clustering heat map of the 28 metabolites. The rows represent the 28 metabolites, and the columns represent samples in the control and ACS patients. VIP scores > 1.0 and FDR < 0.05 were considered as significant differences.

**Figure 4:**
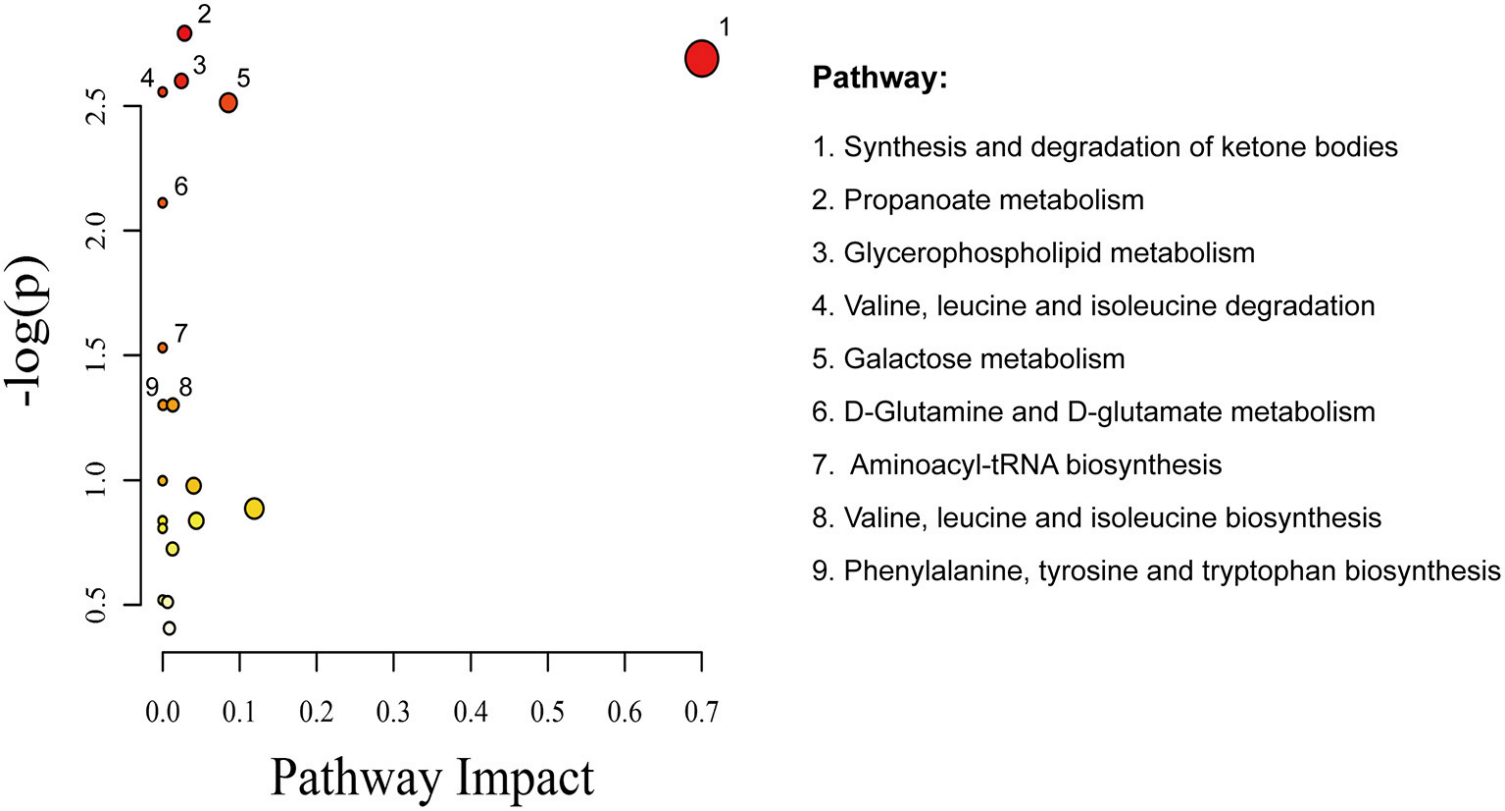
Metabolomics view from pathway analysis performed using MetaboAnalyst. The node color is based on its *p*-value, and the node radius is based on their pathway impact values.

The metabolite features were set as VIP scores > 1.0 and FDR < 0.05. Thereby, a total of 28 significant metabolites were selected that can distinguish ACS patients from control subjects, as shown in Table 2 and Supplementary Table 1. The heat map and hierarchical cluster analysis illustrate the different distribution patterns of a total of 28 potential biomarkers between the two groups, as presented in Figure 3. Next, these differential metabolites were entered as input data to perform the pathway enrichment analysis. The detailed results of the pathway analysis are shown in Figure 4 and Table 3, respectively. The potential pathways were characterized as synthesis and degradation of ketone bodies; phenylalanine metabolism; arginine and proline metabolism; valine, leucine, and isoleucine degradation; and others. These results provided biological relevance between ACS patients and control subjects.

**Table 2:** Summary of differential metabolites to distinguish ACS patients from controls.

**Metabolites**	**M/Z**	**RT (min)**	**Type**	**VIP value**	**Fold change**	***p*-value**
Acetoin	89.060	2.35	[M + H]+	2.73485	0.767718	0.000157
D-fructofuranose	179.056	2.99	[M – H]–	2.43371	1.460485	1.25E-06
LPE(16:0p)	438.296	13.54	[M + H]+	2.06323	1.424668	8.29E-06
Diphenylsulfoxide	203.052	1.45	[M + H]+	1.99240	1.374833	0.00012
5-oxo-D-proline	129.127	3.24	[M]+	1.95935	1.221804	0.000382
3-hydroxy-3-methylglutaric acid	163.039	0.43	[M + H]+	1.94675	1.170112	0.00101
Adenosine	265.949	9.19	[M – H]–	1.83565	0.873886	0.004219
L-cystine	239.127	11.68	[M – H]–	1.82551	0.875852	0.002183
creatinine	114.066	1.14	[M + H]+	1.78853	1.171524	0.001768
Phosphatidylethanolamine lyso 20:4	500.277	12.67	[M – H]–	1.67118	1.259715	0.001234
N-butylbenzenesulfonamide	214.089	10.25	[M + H]+	1.65585	0.938917	0.00273
3-methyl-2-oxovaleric acid	129.056	4.93	[M – H]–	1.57132	1.174449	0.003611
o-toluic acid	137.059	0.43	[M + H]+	1.54001	1.123012	0.003452
8,11-eicosadiynoic acid	303.231	14.86	[M – H]–	1.53945	1.303894	0.003295
LPE(18:2)	478.291	12.75	[M + H]+	1.53936	0.823716	0.015186
L-phenylalanine	164.071	3.54	[M – H]–	1.48969	1.120638	0.003564
O-acetyl-L-Carnitine	238.931	10.43	[M – H]–	1.40231	1.148321	0.009212
2-(2-carboxyethyl)-4-methyl-5-propylfuran-3-carboxylic acid	239.092	9.35	[M – H]–	1.36899	0.779988	0.024651
PC(14:0/0:0)	468.306	12.16	[M + H]+	1.36663	0.850007	0.032162
Acetoacetate	107.967	9.71	[M]+	1.3192	0.897832	0.03983
5-Isopropylbicyclo[3.1.0]hexan-2-one	139.111	0.09	[M + H]+	1.30839	1.119695	0.020492
Galactitol	180.973	4.61	[M – H]–	1.26313	0.8763	0.037811
Indoxylsulfuric acid	212.001	5.26	[M – H]–	1.24185	1.287774	0.025296
Phosphatidylethanolamine lyso 16:0	452.278	13.14	[M – H]–	1.21647	0.86635	0.041439
Choline	104.107	1.11	[M + H]+	1.13647	1.081594	0.045266
LPI(18:2)	595.288	12.64	[M – H]–	1.11604	1.169562	0.041938
Valine	118.086	8.92	[M + H]+	1.06623	1.138983	0.038275
LPC(20:4)	544.337	12.74	[M + H]+	1.01176	1.125107	0.042242

*M/Z, mass to charge ratio; RT, retention time; VIP, variable importance in the projection*.

**Table 3:** The detailed results from the pathway analysis.

**Pathway name**	**Hits/Total**	**Expected**	**Raw *p***	**–log 10(*p*)**	**Holm adjust**	**Impact**
Synthesis and degradation of ketone bodies	1/6	0.069796	0.067866	2.6902	1	0.7
Phenylalanine metabolism	1/45	0.52347	0.41224	0.88614	1	0.11906
Galactose metabolism	2/41	0.47694	0.081006	2.5132	1	0.08543
Fructose and mannose metabolism	1/48	0.55837	0.43291	0.83723	1	0.04372
Butanoate metabolism	1/40	0.46531	0.37618	0.97769	1	0.0403
Propanoate metabolism	2/35	0.40715	0.061325	2.7916	1	0.02848
Glycerophospholipid metabolism	2/39	0.45368	0.074242	2.6004	1	0.02437
Valine, leucine and isoleucine biosynthesis	1/27	0.31408	0.27215	1.3014	1	0.01325
Cysteine and methionine metabolism	1/56	0.65143	0.48465	0.72434	1	0.01289
Purine metabolism	1/92	1.0702	0.66629	0.40603	1	0.00878
Arginine and proline metabolism	1/77	0.89572	0.59972	0.51129	1	0.00645
Phenylalanine, tyrosine and tryptophan biosynthesis	1/27	0.31408	0.27215	1.3014	1	0.00062
Valine, leucine and isoleucine degradation	2/40	0.46531	0.0776	2.5562	1	0
D-Glutamine and D-glutamate metabolism	1/11	0.12796	0.12101	2.1119	1	0
Aminoacyl-tRNA biosynthesis	2/75	0.87246	0.21633	1.5309	1	0
Pantothenate and CoA biosynthesis	1/27	0.31408	0.27215	1.3014	1	0
Nitrogen metabolism	1/39	0.45368	0.36872	0.99773	1	0
Glycine, serine and threonine metabolism	1/48	0.55837	0.43291	0.83723	1	0
Starch and sucrose metabolism	1/50	0.58164	0.44629	0.80678	1	0
Tyrosine metabolism	1/76	0.88409	0.59485	0.51944	1	0

*The “Total” is the total number of metabolites in each pathway; The “Hits” is the actually matched number according to the uploaded data; The “Raw p” is the original P-value calculated from enrichment analysis; The “Holm adjust” is the p-value adjusted by Holm-Bonferroni method and the “Impact” is the pathway impact value calculated from pathway topology analysis*.

### Selection of Potential Biomarkers

**Figure 5:**
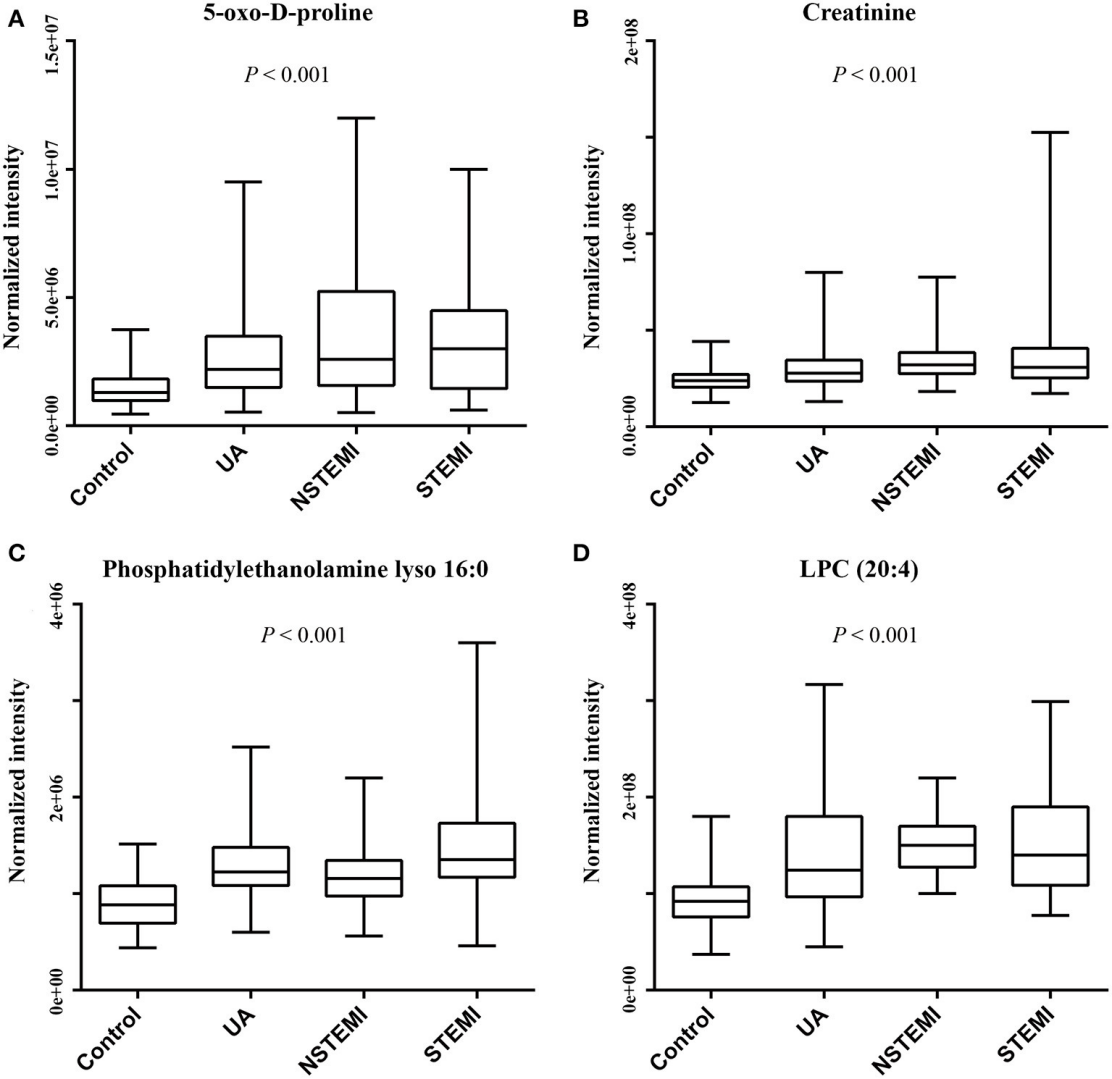
Box plots of metabolites to discriminate ACS patients from controls (ACS patients comprise of UA, NSTEMI, and STEMI patients). **(A)** 5-Oxo-D-proline. **(B)** Creatinine. **(C)** Phosphatidylethanolamine lyso 16:0. **(D)** LPC (20:4).

**Figure 6:**
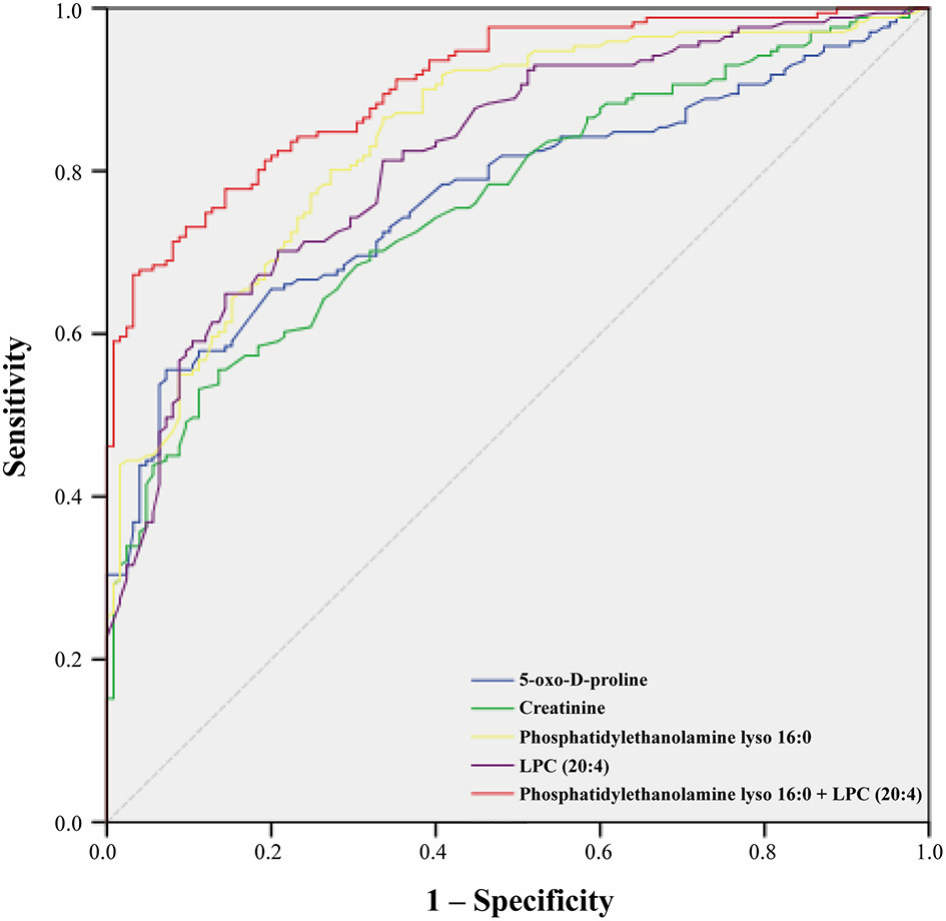
Receiver operating characteristic (ROC) curve model of metabolites to discriminate ACS patients from controls.

On the basis of the above studies, box plots and classical univariate ROC curve analyses were applied to further investigate the clinical diagnostic ability of the metabolite candidates. As would be expected, the box plot represents the relative changes in the four potential metabolites 5-oxo-D-proline, 3-hydroxy-3-methylglutaric acid, phosphatidylethanolamine lyso 16:0, and LPC (20:4) which differ significantly among subclinical types of ACS patients and controls (Figure 5). Furthermore, ROC curves also showed an apparent discrimination of these four metabolites between groups, as shown in Figure 6 and Table 4. Among them, the ROC curves of phosphatidylethanolamine lyso 16:0 and LPC (20:4) showed a relatively higher diagnostic performance from controls with AUC values of 0.844 (95% CI: 0.800–0.888; *p* < 0.001) and 0.821 (95% CI: 0.774–0.868; *p* < 0.001), respectively, while the ROC curves of 5-oxo-D-proline and creatinine exhibited a moderate diagnostic performance with AUC values of 0.772 (95% CI: 0.720–0.825; *p* < 0.001) and 0.764 (95% CI: 0.711–0.817; *p* < 0.001), respectively. In addition, the combination of phosphatidylethanolamine lyso 16:0 and LPC (20:4) significantly improved the AUC value of 0.905 (95% CI: 0.873–0.937; *p* < 0.001) than in single metabolite detection mode.

**Table 4:** Clinical value of metabolic biomarkers detection to diagnosis of ACS.

**Metabolites**	**AUC**	**Standard error**	**95% CI**	***p*-value**
5-oxo-D-proline	0.772	0.027	0.720–0.825	<0.001
Creatinine	0.764	0.027	0.711–0.817	<0.001
Phosphatidy-lethanolamine lyso 16:0	0.844	0.022	0.800–0.888	<0.001
LPC (20:4)	0.821	0.024	0.774–0.868	<0.001
Phosphatidy-lethanolamine lyso 16:0 + LPC (20:4)	0.905	0.016	0.873–0.937	<0.001

*AUC, area under curve; CI, confidence interval; ROC, receiver operating characteristic*.

## Discussion

Metabolomics could be of great advantage in the study of metabolite profiling in human disease, where metabolism is changeable in the biochemical activities of an organism (7, 13, 26). Plasma metabolomics analysis can provide a more comprehensive understanding of the metabolite alterations in ACS (10, 27). Herein, an untargeted LC-MS-based metabolomics profiling platform was conducted to analyze the changes of plasma metabolite levels in 284 ACS patients and 130 controls. The presented results demonstrate the metabolic level in ACS patients differing from those of controls. Particularly, 28 significantly changed metabolites were determined and subsequent metabolic pathway analysis showed that these altered metabolites were mainly involved in several biochemical pathways, including synthesis and degradation of ketone bodies, phenylalanine metabolism, and arginine and proline metabolism. Furthermore, the univariate ROC curve revealed that the existence of four metabolites can correctly distinguish ACS patients from the control group at a certain extent, respectively. Within the limits of our untargeted metabolomics approach, we postulate that a subset of differentially abundant metabolites can serve as potential ACS biomarkers and may provide corroborating evidence of the involvement of plasma metabolite in the pathogenesis of ACS.

The atherosclerotic processes are often present for years before developing into the onset of overt disease; there has been great interest in determining whether metabolomic profiling can identify individuals for the development of subclinical atherosclerosis. By design, in the present research, plasma metabolite profiling of ACS patients revealed differences with those of healthy controls. These results were in concordance with other recently published data describing abnormal metabolites in CAD. For example, of these metabolites, changes in serum creatinine are known to contribute to the incidence of CAD independent of traditional risk factors (26). The ketone body acetoacetate is an energy-providing substrate of respiration of the heart, but it varies with the pathophysiological state (31, 32). In spite that the underlying mechanisms for these changes remain unclear, it could be speculated that the pathological process of ACS is related with slower metabolism and less energy expenditure requirements. Choline has various metabolic roles, and experimental evidence clearly indicates that the choline moiety in dietary phosphatidylcholine (PC) could produce proatherogenic species after metabolism by intestinal microbiota (13, 33, 34). Consistently, other changes are in lysophosphatidylcholine (LPC) and lysophosphatidylethanolamine (LPE), which are lipid classes known to contribute to the risk of CVD (35, 36). Several prior studies also have demonstrated that LPC is one of the major by-products of phospholipid oxidation, and it may be an implication for atherosclerotic plaque characteristics (36–38). Collectively, this consistency reinforces the potential causal relationship between significantly altered concentrations of metabolites and ACS, and the relatively subtle perturbations in metabolic might serve as potential diagnostic biomarkers in patients with ACS.

Additionally, observations of changes in aberrant metabolites in the setting of ACS in fact reflected coordinate changes in several important metabolic pathways. For instance, among these metabolic pathways, synthesis and degradation of the ketone body pathway were identified as vital pathways involved in the pathogenesis of atherosclerosis, which have been well-characterized and widely studied (39, 40). Phenylalanine is an essential aromatic amino acid and the precursor for tyrosine and dopamine-related neurotransmitters. A small case–control study using LC-MS suggested that an amino acid score including phenylalanine is associated with cardiovascular risk (17). Subsequent metabolite profiling in a large prospective cohort has also affirmed this conclusion (14). Valine has been reported to be associated with metabolic risk factors, insulin resistance, incident type 2 diabetes, and future cardiovascular events (41, 42). Recently, it has been reported that arginine is crucial for nitric oxide (NO) synthesis and the bioavailability of NO is particularly involved in endothelium dysfunction, eventually leading to an increased risk of cardiovascular mortality (43). A previous metabolomics study has revealed that branched-chain amino acid concentrations were independently associated with death or myocardial infarction during follow-up (44). Altogether, these results illustrate the potential importance metabolic pathways in ACS, as function and metabolism are inextricably linked. Further studies are required to understand the mechanistic significance of these altered metabolites in cardiovascular disease.

In addition, we examined the discriminative capability of the metabolites identified in our study using ROC analysis. Indeed, the current clinical symptoms from the differential diagnosis of ACS have been a bottleneck to solve because of the interindividual variability (7, 45). Of interest, in the present study, groups were apparently differentiated depending on four candidate metabolites (5-oxo-D-proline, creatinine, phosphatidylethanolamine lyso 16:0, and LPC (20:4), with an AUC range from 0.764 to 0.821. The combined analysis of phosphatidylethanolamine lyso 16:0 and LPC (20:4) was interesting and potentially applicable for improved identification of ACS patients. Although our study shows that single metabolites could be used as biomarkers to differentiate between controls and ACS patients, and a combination of these metabolites may improve predictive performance (46), it remains to be determined in clinical trials.

Prior substantial studies have identified many potential metabolites with CAD in a wide range of population; however, results are not always unequivocal. It is important to recognize that the clinical manifestations vary greatly among ACS patients, ranging from unstable angina without myocardial necrosis to ST segment elevation myocardial infarction. Metabolites obtained from patients with ACS may be very promiscuous, and no single analysis or method is adequate to reliably identify all of these metabolites in human samples. For example, a prior study analyzed peripheral plasma from non-ST segment elevation ACS patients, suggesting that the panel of biomarkers consisting of 5-OH-tryptophan, 2-OH-butyric acid, and 3-OH-butyric have a great potential for early diagnosis of ACS (47). Another recent study comprehensively analyzed the serum metabolomic change in ACS patients, proposing that the combined model of serum betaine and ejection fraction might serve as a potential diagnostic biomarker for the vulnerability of plaque stability (48). In this study, we also successfully characterized the significantly altered metabolites using blood samples in the setting of ACS. However, our findings are not entirely consistent with previous studies. The results of these studies indicated that serum metabolite profiling of ACS patients revealed a myriad of differentially occurring metabolites. The heterogeneity of the results might relate to differences in age, sex, ethnicity, geographical region, nutritional intake, medications, and lifestyle factors, as well as differences in analytical methods, which are known to influence metabolite levels (49). These characteristics are particularly important when analyzing the complex mixtures of metabolites.

### Study Limitations

Several limitations of our study should be noted. Firstly, it must be mentioned that, due to the one single cohort and the choice for an untargeted metabolomics approach, the results and conclusions drawn to these metabolites should be interpreted with caution. Therefore, our findings should be validated in a larger population, as well as in other regions and ethnic groups. Secondly, the cross-sectional design rather than longitudinal design of the study may limit the clinical value of the measured metabolites. Hence, additional prospective validation is required to judge the clinical usefulness of these presented metabolites in pretreatment and post-treatment samples in ACS patients.

## Conclusion

In conclusion, this study provided relatively wider metabolite coverage in patients with ACS by using an untargeted LC/MS metabolomics profiling approach. The apparent differences may reflect the onset and progression of atherosclerosis in ACS and could lead to novel diagnostic biomarker development and clinical application. However, these results are explorative and further investigation with external validation is warranted to underpin these observations and specify their role in ACS-specific metabolic alteration.

## Data Availability Statement

The datasets used and analyzed during the current study are available from the corresponding author on reasonable request.

## Ethics Statement

The studies involving human participants were reviewed and approved by Medical Ethics Committee of the Meizhou People's Hospital. The patients/participants provided their written informed consent to participate in this study.

## Author Contributions

WZ, ZZ, and JH contributed to the study concept and design, conducted the literature search, and wrote the manuscript. WZ, QD, and XD contributed to the data analysis and made the tables and figures. QD and XD contributed to the collection of patients' samples and medical information. ZZ and JH critically revised the manuscript. All authors contributed to the article and approved the submitted version.

## Conflict of Interest

The authors declare that the research was conducted in the absence of any commercial or financial relationships that could be construed as a potential conflict of interest.

## Supplementary Material

Supplementary Figure 1 Representative LC-MS chromatograms of plasma derived from controls and ACS patients.

Supplementary Table 1 Summary of the molecular features of differential metabolites between ACS patients and controls.
