# Supplementary figures and images for: Plasma Metabolomics of Acute Coronary Syndrome Patients Based on Untargeted Liquid Chromatography–Mass Spectrometry
Source: Front Cardiovasc Med. 2021 May 20;8:616081. doi: 10.3389/fcvm.2021.616081 (PMC8172787; doi:10.3389/fcvm.2021.616081)

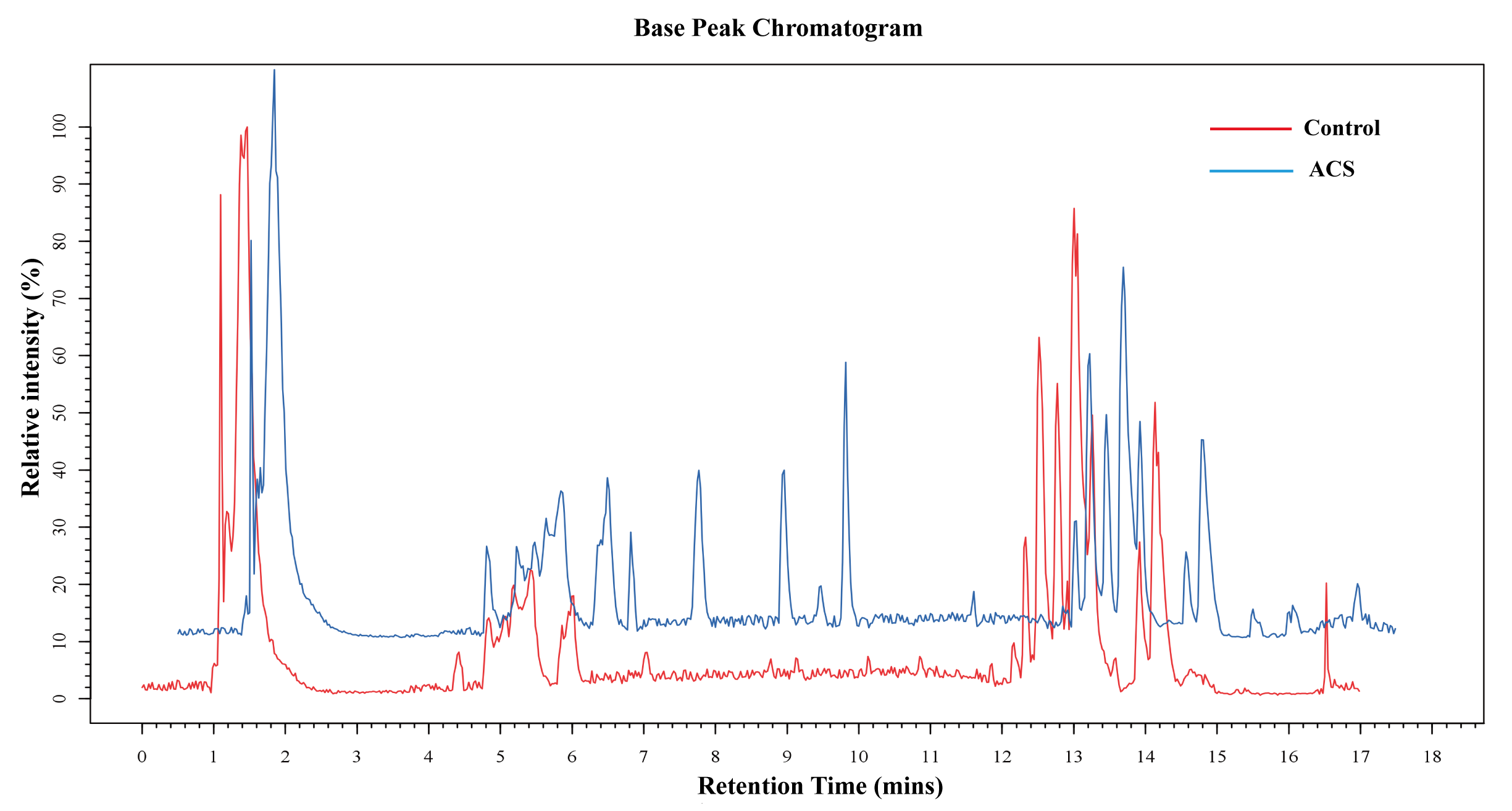

Supplement: Supplementary Figure 1 — Representative LC-MS chromatograms of plasma derived from controls and ACS patients. [file Image_1.TIF]
